# Inverse Correlation between Vitamin D and C-Reactive Protein in Newborns

**DOI:** 10.3390/nu7115468

**Published:** 2015-11-10

**Authors:** Rui-Xue Tao, Qi-Fan Zhou, Zhi-Wei Xu, Jia-Hu Hao, Kun Huang, Zhe Mou, Xiao-Min Jiang, Fang-Biao Tao, Peng Zhu

**Affiliations:** 1 Department of Gynecology and Obstetrics, Hefei First People’s Hospital, Hefei 230001, China; taoruixue.good@163.com; 2 Department of Maternal, Child & Adolescent Health, and Anhui Provincial Key Laboratory of Population Health & Aristogenics, Anhui Medical University, Hefei 230032, China; zhouqifan8023@163.com (Q.-F.Z.); jia0556@163.com (J.-H.H.); wuweihk8028@163.com (H.K.); fbtao@ahmu.edu.cn (F.-B.T.); 3 School of Public Health and Social Work, and Institute of Health and Biomedical Innovation, Queensland University of Technology, Brisbane 4059, Australia; xzw1011@gmail.com; 4 Shanghai Key Laboratory of Meteorology and Health, Shanghai 200001, China; zhem@163.com; 5 Department of Gynecology and Obstetrics, Hefei Maternal and Child Health Hospital, Hefei 230001, China; jxm8080@sina.com

**Keywords:** 25-hydroxyvitamin D, vitamin D, C-reactive protein, inflammation, neonate

## Abstract

Some studies suggested that adequate vitamin D might reduce inflammation in adults. However, little is known about this association in early life. We aimed to determine the relationship between cord blood 25-hydroxyvitamin D (25(OH)D) and C-reactive protein (CRP) in neonates. Cord blood levels of 25(OH)D and CRP were measured in 1491 neonates in Hefei, China. Potential confounders including maternal sociodemographic characteristics, perinatal health status, lifestyle, and birth outcomes were prospectively collected. The average values of cord blood 25(OH)D and CRP were 39.43 nmol/L (SD = 20.35) and 6.71 mg/L (SD = 3.07), respectively. Stratified by 25(OH)D levels, per 10 nmol/L increase in 25(OH)D, CRP decreased by 1.42 mg/L (95% CI: 0.90, 1.95) among neonates with 25(OH)D <25.0 nmol/L, and decreased by 0.49 mg/L (95% CI: 0.17, 0.80) among neonates with 25(OH)D between 25.0 nmol/L and 49.9 nmol/L, after adjusting for potential confounders. However, no significant association between 25(OH)D and CRP was observed among neonates with 25(OH)D ≥50 nmol/L. Cord blood 25(OH)D and CRP levels showed a significant seasonal trend with lower 25(OH)D and higher CRP during winter-spring than summer-autumn. Stratified by season, a significant linear association of 25(OH)D with CRP was observed in neonates born in winter-spring (adjusted β = −0.11, 95% CI: −0.13, −0.10), but not summer-autumn. Among neonates born in winter-spring, neonates with 25(OH)D <25 nmol/L had higher risk of CRP ≥10 mg/L (adjusted OR = 3.06, 95% CI: 2.00, 4.69), compared to neonates with 25(OH)D ≥25 nmol/L. Neonates with vitamin D deficiency had higher risk of exposure to elevated inflammation at birth.

## 1. Introduction

Considerable evidence has linked early-life vitamin D deficiency with increased risk for fetal growth restriction, infection, and poor neurodevelopment in later life [1,2,3]. Similarly, research has shown that fetal exposure to elevated inflammation is associated with adverse health outcomes including fetal growth restriction, infection, and abnormal brain development [4,5,6]. Therefore, early-life vitamin D deficiency may impact offspring development through an inflammatory pathway.

Several studies *in vitro* have suggested that vitamin D supplementation inhibits proinflammatory cytokine production in various cell lines including monocytes and macrophages [7,8]. C-reactive protein (CRP) is a nonspecific marker for persisting inflammatory states. Neonatal CRP >10 mg/L was suggested to be strongly correlated to several adverse clinical consequence such as sepsis [9,10] and chorioamnionitis [11,12]. Elevated CRP is also associated with cognitive impairment [13], cardiovascular disease [14], and diabetes mellitus [15] in adults. A cross-sectional study with a large sample suggested an inverse relationship between vitamin D and CRP in asymptomatic adults with low vitamin D levels [16]. However, randomized controlled trials assessing the effectiveness of vitamin D supplementation in reducing CRP showed inconsistent results [17,18,19].

To date, little is known about the association between vitamin D and CRP in newborn. It has been confirmed that 25(OH)D readily crosses the placenta [20]. However, CRP does not cross the placenta [21]. Consequently, the fetal CRP response to mediators including 25(OH)D is independent of the maternal response [22]. Given the high prevalence of vitamin D deficiency among pregnant women worldwide [23,24], it is important to confirm the association between vitamin D and informatory response in fetus/neonates.

In this prospective observational study with a large sample, we assessed the association between 25(OH)D and CRP in cord blood at birth. Stratifications by 25(OH)D levels and seasons were performed to further examine the associations.

## 2. Methods

### 2.1. Patients and Study Design

2552 pregnant women, of gestational ages from 30 to 34 weeks, were recruited in Hefei (32° N latitude) from January to September 2008. Participants completed a structured questionnaire, including sociodemographic characteristics, perinatal lifestyle, and pregnancy history. Nurses collected the information needed from the participants using questionnaires through face-to-face interviews. Pregnancy complications, delivery outcomes were obtained through the medical records. Cord blood sample at birth was collected randomly when available. The exclusion criterion was stillbirth, birth defect, delivery before 32 weeks of gestation, pregnancy with assisted reproductive technology, multiple gestations, pregnant women with acute infections, surgery, and trauma during the previous three months, or newborns whose 5 min Apgar score below 7. Finally, we obtained available data from 1491 mother-infant pairs with cord blood sample. Ethics committee approval for the study was obtained from the ethics committees of Anhui Medical University, and informed consent was obtained from each participant.

### 2.2. Cord Blood 25-Hydoxyvitamin D and C-Reactive Protein

After delivery, cord blood sample was immediately collected and anticoagulated by use of sodium heparin. Plasma sample was centrifuged and promptly refrigerated at −4 °C, within 12 h, and then it was transferred to −80 °C freezers for long-term storage. Plasma concentrations of 25(OH)D and CRP were measured using the commercial radioimmunoassay (RIA) kits (DiaSorin, Stillwater, MN, USA) and the enzyme-linked immunosorbent assay (ELISA) kits (Cusabio Biotech, Wuhan, China) according to manufacturer’s instructions respectively. Both intra-assay coefficients of variation were less 10%. Plasma concentrations of 25(OH)D were analyzed as continuous variable, decile, and categorical variables with cutoffs of 25 nmol/L and 50 nmol/L as recommended by the Canadian Paediatric Society [25]. Vitamin D deficiency was defined as 25(OH)D concentrations less than 25 nmol/L. Plasma concentrations of CRP were analyzed both as continuous variable and categorical variable with cutoffs of 6 mg/L and 10 mg/L [10].

### 2.3. Potential Confounders

Information on potential confounders, including maternal sociodemographic characteristics, perinatal health status, lifestyle, birth outcomes and seasons, was prospectively collected from medical records or interviews. Maternal sociodemographic characteristics included age, education attainment (≤9 or >9 years of completed schooling) and income (<2000 or ≥2000 RMB Yuan/month). Perinatal health status included parity (nulliparae or multiparae), prepregnancy body mass index (BMI), gestational weight gain (GWG), and pregnancy complications. Pregnancy complications included moderate or severe anemia, hypertension, diabetes mellitus, glandula thyroidea disease, abnormal heart function, and intrahepatic cholestasis of pregnancy.

Perinatal lifestyle included maternal alcohol consumption (none or any), paternal smoking (less or more than 6 cigarettes/day), and alcohol consumption (none or any) during up to six months before pregnancy, and maternal vitamin D supplementation (less or more than two months) during pregnancy. Birth outcomes included gestational age at birth, birth weight, infant gender, mode of delivery, and birth season. The gestational age at birth based on the difference between the date of the last menstrual period and the date of delivery and was categorized as <37 (preterm birth) or ≥37 gestational weeks. Low birth weight was defined as neonatal weight less than 2500 g. Small for gestational age (SGA) was defined as birth weight <10th percentiles of distribution for gestational age and infant sex [26]. Season was designated as winter (December, January, February), spring (March, April, May), summer (June, July, August), or autumn (September, October, November).

### 2.4. Statistical Analysis

General linear regression models and Mantel-Haenszel chi-square test were used to compare means and proportions for the characteristics of mother-infant pairs according to cord blood 25(OH)D and CRP levels, respectively. The means of cord blood CRP and the proportions for CRP ≥10 mg/L across deciles of 25(OH)D levels were presented. The differences in the means of CRP levels and the risk of CRP ≥10 mg/L across deciles of 25(OH)D level were examined using multiple linear regression models and logistic regression models, respectively. The crude regression coefficients (β) and odd ratios (OR), and adjusted β and OR were generated, with 95% confidence interval (CI). Potential confounders adjusted in multiple regression models included maternal sociodemographic characteristics, perinatal health status, lifestyle, and birth outcomes.

We further assessed the adjusted associations between 25(OH)D and CRP levels among different 25(OH)D levels (<25.0 nmol/L, 25.0–49.9 nmol/L and ≥50 nmol/L), using multiple linear models with adjustment for potential confounders. A non-linear trend in cord blood CRP levels across 25(OH)D levels was observed. Then, the non-linear relations of cord blood 25(OH)D levels and CRP were fitted in nonlinear regression models using a sine function.

Seasonality of cord blood 25(OH)D and CRP was tested by fitting the data to linear model and nonlinear model using sine function, respectively. The goodness-of-fit of nonlinear models were checked for adequacy. Stratified by season (winter-spring or summer-autumn), the adjusted linear relations between cord blood 25(OH)D and CRP were examined using multiple linear models after adjusting for potential confounders. The adjusted difference on CRP levels and adjusted OR of CRP ≥10 mg/L for neonates with 25(OH)D <25 nmol/L were generated, compared to neonates with 25(OH)D ≥25 nmol/L. All statistical analyses were performed using SPSS statistical software (version 21.0, IBM Corp: Armonk, NY, USA).

## 3. Results

The characteristics of 1491 mother-infant pairs in this study were presented in Table 1. The mean cord blood 25(OH)D and CRP concentrations were 39.43 nmol/L (standard deviation (SD): 20.35, range: 6.06–119.64) and 6.71 mg/L (SD: 3.07, range: 0.24–22.42), respectively. Cord blood 25(OH)D concentrations were higher in newborns of women who took vitamin D supplements and in those born in summer or autumn. Gestational weight gain was negatively associated with cord blood 25(OH)D concentration. With the increase of CRP concentrations, the means of maternal age, gestational weeks, and birth weight significantly decreased, the proportions of maternal vitamin D supplementation and birth in summer-autumn significantly decreased, and the proportion of SGA significantly increased.

**Figure 1:**
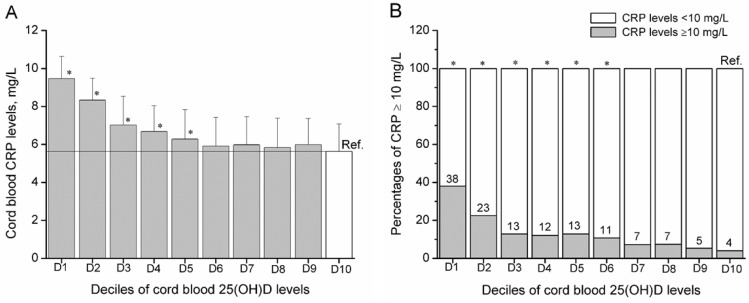
Means of cord blood CRP levels (**A**) and proportions of CRP ≥10 mg/L (**B**) across deciles of 25(OH)D levels in neonates (*n* = 1491). Bars represent mean ± SD (**A**). D1, D2 and D3 mean the first, second and third decile respectively, and so on. ∗ Represents the significant difference with *p* < 0.05, compared to the tenth decile of 25(OH)D levels.

The means of CRP levels (*p*-trend < 0.001) and proportions of CRP ≥10 mg/L (*p*-trend < 0.001) significantly decreased across deciles of 25(OH)D concentrations. Compared to neonates in the 10th decile of 25(OH)D concentrations, neonates in the first (adjusted β = 3.71, 95% CI: 3.10, 4.32; adjusted OR = 9.49, 95% CI: 3.56, 25.32), second (adjusted β = 2.52, 95% CI: 1.91, 3.14; adjusted OR = 5.26, 95% CI: 1.96, 14.08), third (adjusted β = 1.42, 95% CI: 0.75, 2.09; adjusted OR = 2.82, 95% CI: 1.03, 7.73), fourth (adjusted β = 1.01, 95% CI: 0.35, 1.69; adjusted OR = 2.96, 95% CI: 1.09, 8.01) and fifth (adjusted β = 0.99, 95% CI: 0.29, 1.69; adjusted OR = 3.21, 95% CI: 1.20, 8.59) deciles of 25(OH)D had higher CRP concentrations (Figure 1A) and higher risk of CRP ≥10 mg/L (Figure 1B).

**Figure 2:**
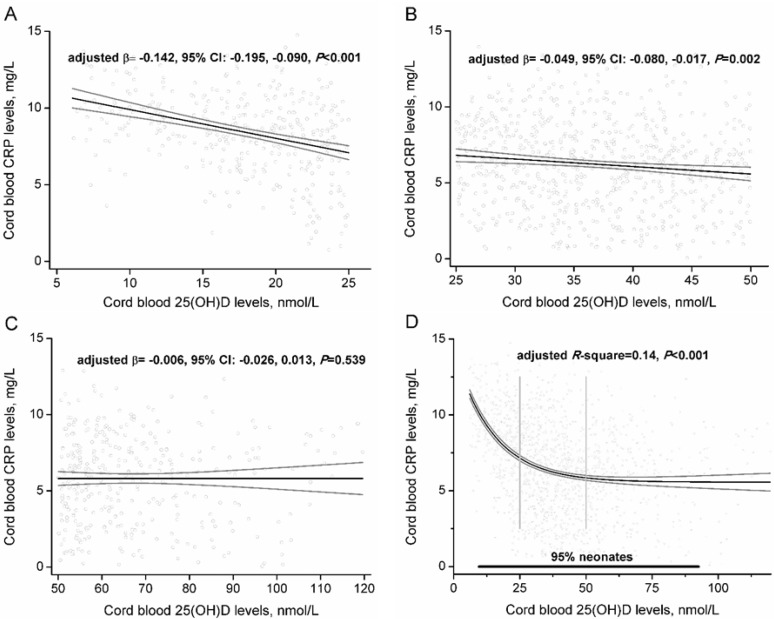
Association between cord blood 25(OH)D and CRP levels, stratified by 25(OH)D levels. Multiple linear models were conducted to assessed the adjusted regression coefficient among neonates with 25(OH)D less than 25.0 nmol/L (**A**, *n* = 389), between 25.0 nmol/L and 49.9 nmol/L (**B**, *n* = 731) and more than 50.0 nmol/L (**C**, *n* = 371), respectively. Potential confounders included maternal sociodemographic characteristics, perinatal health status, lifestyle, and birth outcomes. A nonlinear regression model with sine function was conducted to fit the nonlinear relation between 25(OH)D and CRP among all neonates (**D**, *n* = 1491). The solid black line denotes the fit of the regression model, the solid grey line denote 95% CI. The vertical lines denote 25(OH)D benchmarks.

Stratified by 25(OH)D concentrations, per 10 nmol/L increase in 25(OH)D levels, CRP decreased by 1.42 mg/L (95% CI: 0.90, 1.95) in neonates with 25(OH)D <25.0 nmol/L (Figure 2A) and decreased by 0.49 mg/L (95% CI: 0.17, 0.80) in neonates with 25(OH)D between 25.0 nmol/L and 49.9 nmol/L (Figure 2B), after adjusting for potential confounders. However, no significant association between 25(OH)D and CRP was observed in neonates with 25(OH)D ≥50 nmol/L (Figure 2C). A non-linear trend in CRP levels across 25(OH)D concentrations was observed. We further fitted the non-linear regression model using a sine function to best display the association. The solid black line shows the best fit analysis for the association of 25(OH)D with the CRP (adjusted *R*-square = 0.14, *p* < 0.001) (Figure 2D). The model fitted the data well as the residuals were randomly distributed.

**Table 1:** Characteristics of mother-infant pairs according to cord blood 25(OH)D and CRP levels.

Characteristics	All Subjects (*n* = 1491)	25(OH)D Levels (nmol/L)			*p*-Values *	CRP Levels (mg/L)			*p*-Values *
		<25.0 (*n* = 389)	25.0–49.9 (*n* = 731)	≥50.0 (*n* = 371)		<6.0 (*n* = 577)	6.0–9.99 (*n* = 716)	≥10.0 (*n* = 198)	
**Sociodemographic characteristics**									
Maternal age, year, mean (SD)	27.65 (3.66)	27.4 (3.5)	27.8 (3.7)	27.6 (3.7)	0.441	27.9 (3.7)	27.6 (3.7)	27.1 (3.3)	0.005
Maternal education <9 years, *n* (%)	319 (21)	85 (22)	146 (20)	79 (21)	0.840	115 (20)	147 (21)	48 (24)	0.265
Maternal income <2000 RMB yuan/month, *n* (%)	225 (15)	65 (17)	105 (14)	55 (15)	0.461	82 (14)	109 (15)	34 (17)	0.324
**Perinatal health status**									
Prepregnancy BMI, kg/m^2^, mean (SD)	20.16 (2.41)	20.3 (2.6)	20.1 (2.4)	20.2 (2.3)	0.739	20.1 (2.2)	20.2 (2.6)	20.0 (2.2)	0.793
GWG, kg, mean (SD)	16.76 (4.85)	17.0 (4.9)	17.0 (4.9)	16.1 (4.6)	0.007	16.7 (4.7)	16.9 (4.9)	16.7 (5.1)	0.531
Multipara, *n* (%)	199 (13)	50 (13)	104 (14)	45 (12)	0.782	84 (15)	93 (13)	22 (11)	0.198
Pregnancy complications ^a^, *n* (%)	226 (15)	72 (19)	102 (14)	52 (14)	0.081	81 (14)	114 (16)	31 (16)	0.426
**Lifestyle** ^b^									
Maternal alcohol consumption ^c^, *n* (%)	225 (15)	59 (15)	116 (16)	50 (14)	0.524	90 (16)	110 (15)	25 (13)	0.403
Paternal alcohol consumption ^c^, *n* (%)	1199 (80)	308 (79)	576 (79)	315 (85)	0.050	462 (80)	586 (82)	151 (76)	0.547
Paternal smoking ^d^, *n* (%)	336 (23)	91 (23)	166 (23)	79 (21)	0.490	123 (21)	158 (22)	55 (28)	0.110
Vitamin D supplementation ^e^, *n* (%)	712 (48)	171 (44)	344 (47)	197 (53)	0.012	287 (50)	345 (48)	80 (40)	0.046
**Birth outcomes**									
Female infant, *n* (%)	700 (47)	198 (51)	338 (46)	164 (44)	0.063	258 (45)	342 (48)	100 (51)	0.125
Cesarean section, *n* (%)	842 (57)	204 (52)	428 (59)	210 (57)	0.237	334 (58)	405 (57)	103 (52)	0.189
Birth during summer or autumn ^f^, *n* (%)	792 (53)	40 (10)	429 (59)	323 (87)	<0.001	348 (60)	387 (54)	57 (29)	<0.001
Gestational weeks, week, mean (SD)	38.91 (1.46)	38.8 (1.7)	39.0 (1.4)	38.9 (1.4)	0.253	38.98 (1.34)	38.93 (1.45)	38.64 (1.88)	0.014
Birth weight, g, mean (SD)	3385 (450)	3320 (503)	3437 (417)	3348 (440)	0.353	3463 (409)	3378 (444)	3181 (516)	<0.001
SGA, *n* (%)	133 (9)	50 (13)	46 (6)	37 (10)	0.147	25 (4)	61 (9)	47 (24)	<0.001

CRP, C-reactive protein; GWG, gestational weight gain; SGA, small for gestational age. ^a^ Pregnancy complications included moderate or severe anemia, hypertension, diabetes mellitus, glandula thyreoidea disease, abnormal heart function and intrahepatic cholestasis of pregnancy; ^b^ Prepregnancy lifestyle means lifestyle during up to six months before pregnancy; ^c^ Alcohol consumption was defined as any alcohol consumption; ^d^ Father’s smoking was defined as more than 6 cigarettes daily; ^e^ Maternal vitamin D supplementation was limited to those who taking vitamin D supplementation more than two months during pregnancy; ^f^ Summer (June, July, August) and autumn (September, October, November). * Test for trend based on Mantel-Haenszel chi-square test for categorical variables and linear regression for continuous variables.

**Figure 3:**
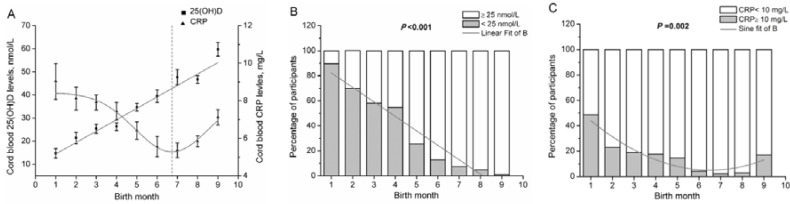
Seasonality of cord blood 25(OH)D and CRP (*n* = 1491). The trends of cord blood 25(OH)D and CRP levels across birth month were assessed by fitting the data to the best-fitting linear and sinusoidal model, respectively (**A**). The proportions of cord blood 25(OH)D <25.0 nmol/L (**A**) and CRP ≥ 10 mg/L (**B**) across birth month were assessed by linear regression model and nonlinear regression with sine function, respectively. Bars represent mean and 95% CI of the mean (**A**). The solid grey line denotes the fit of the regression model (**A**–**C**). The vertical lines denote the birth month of fitted minimum concentrations of CRP (**A**).

Neonates born in winter-spring had significant lower means of 25(OH)D (27.59 ± 13.30 nmol/L *vs.* 49.89 ± 19.78 nmol/L, *p* < 0.001) and higher means of CRP (7.40 ± 3.21 mg/L *vs.* 6.19 ± 2.81 mg/L, *p* < 0.001), compared to neonates born in summer-autumn. To explore the seasonality of 25(OH)D and CRP levels, linear model (adjusted *R*-square = 0.96, *p* < 0.001) and nonlinear model using sine function (adjusted *R*-square = 0.97, *p* < 0.001) were performed to fit the 25(OH)D and CRP concentrations across birth month, respectively. Fitted maximum concentrations of CRP and minimum concentrations of 25(OH)D were simultaneously observed in neonates born in January. They also had maximum proportions of 25(OH)D <25 nmol/L and CRP ≥10 mg/L. Fitted maximum concentrations of 25(OH)D were observed in neonates born in September, however, fitted minimum concentrations of CRP and proportions of CRP ≥10 mg/L were observed in neonates born in June, rather than September (Figure 3). Additionally, Figure 3A suggested that there was a positive relation between 25(OH)D and CRP across birth month in neonates with 25(OH)D ≥42 nmol/L and born between July to September. However, multiple linear model showed no significant association after adjusting for potential confounders.

**Figure 4:**
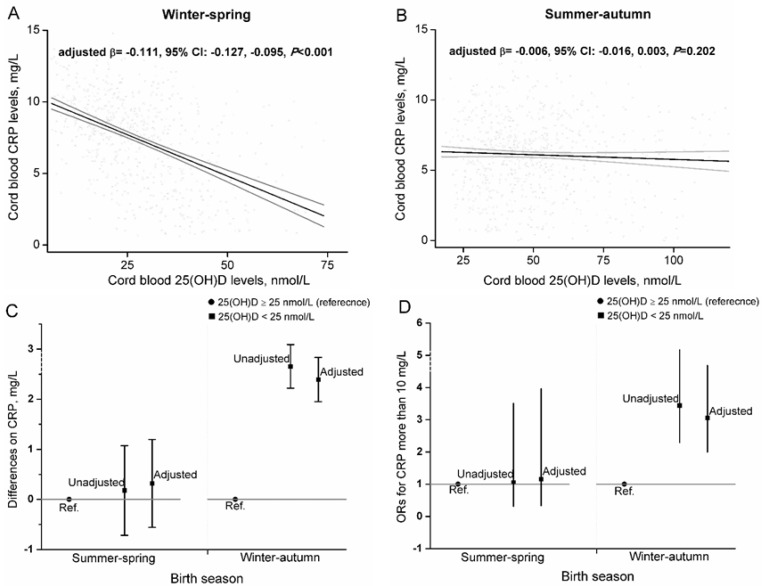
Associations between cord blood 25(OH)D and CRP, stratified by season. The adjusted linear relation was assessed by multiple linear models in neonates born in winter-spring (**A**, *n* = 699) and summer-autumn (**B**, *n* = 792). The solid black line denotes the fit of the regression model, the solid grey line denote 95% CI (**A**,**B**). The adjusted differences on CRP levels (**C**) and adjusted ORs of CRP ≥10 mg/L (**D**) for neonates with 25(OH)D <25 nmol/L were assessed by multiple linear model. Potential confounders included maternal sociodemographic characteristics, perinatal health status, lifestyle, and birth outcomes. Bars represent regression coefficients (**C**) or ORs (**D**), with 95% CI.

Stratification by season was performed to further explore the association of 25(OH)D with CRP. A significant linear association of 25(OH)D with CRP was observed in neonates born in winter-spring, but not summer-autumn. CRP decreased by 1.11 mg/L (95% CI: 0.95, 1.27) per 10 nmol/L increase in 25(OH)D among neonates born in winter-spring after adjusted for potential confounders. Accordingly, neonates with 25(OH)D <25 nmol/L had higher CRP concentrations (adjusted β = 2.39, 95% CI: 1.952, 2.837) and higher risk of CRP ≥10 mg/L (adjusted OR = 3.06, 95% CI: 2.00, 4.69), compared to neonates with 25(OH)D ≥25 nmol/L, when they were born in winter-spring (Figure 4).

## 4. Discussion

In this observational study based on prospective birth cohort, we observed an inverse relation between cord blood vitamin D and CRP in neonates with 25(OH)D <50 nmol/L, especially <25 nmol/L. The inverse relation was found in neonates born in winter-spring, but not summer-autumn. The findings in our study suggested that fetal vitamin D deficiency was significantly associated with the exposure to higher inflammation at birth.

Vitamin D status has been suggested to be inversely associated with CRP in asymptomatic [16], elderly [27], obese [28] adults and children with lupus [29] in previous cross-sectional studies. The finding in this study is consistent with previous studies. However, prior randomized controlled trials assessing the effectiveness of vitamin D supplementation in reducing CRP found inconsistent results [17,18,19]. Recently, a meta-analysis of 10 trials involving a total of 924 participants showed that vitamin D supplementation significantly decreased the circulating CRP levels in adults [30]. Another trial showed that vitamin D supplementation during pregnancy resulted in a significant decrease in maternal serum CRP [31]. If the inverse relation between vitamin D and CRP in neonates with low vitamin D levels in this study is causality, the findings raised a possibility of protection against excessive inflammatory response in the fetus through vitamin D supplementation among pregnant women with vitamin D deficiency. Further interventional trials are needed to confirm the potential protective effect of adequate vitamin D on fetal inflammatory response.

Seasonality of cord blood vitamin D, CRP, and the association between one another observed in this study was interesting. In humans, circulating vitamin D is mainly derived from synthesis in the skin under the influence of sunlight, depending on season and latitude [32]. Consequently, cord blood vitamin D is subject to the seasonality of maternal vitamin D [33,34]. Few previous studies have revealed the seasonality of CRP levels with higher values during winter and spring than in summer [35,36], which is similar to our finding. However, the mechanisms underlying CRP seasonal variations remain unknown, though the high CRP levels might be induced by respiratory infections in winter-spring [37]. In this study, our findings suggested a hypothesis that seasonality of CRP levels might be mainly depending on the seasonal variations of vitamin D, as the association of vitamin D with inflammation [7,38].

Additionally, we found that 25(OH)D at a level ≥50 nmol/L was not associated with a decrease in CRP, and neonates born in September had maximum levels of vitamin D, but had not minimum levels of CRP accordingly. It appears that high fetal vitamin D levels might have no additional effects on the protection of inflammation, even pro-inflammatory effect, which was supported by previous studies. The U-shaped association between 25(OH)D and CRP indicates that ever-increasing 25(OH)D concentrations may also be related to proinflammatory states [16,39]. The difference on the association of vitamin D with CRP between low and high 25(OH)D levels probably explained the finding that the inverse relation between vitamin D and CRP was observed in neonates born in winter-spring, but not summer-autumn.

To the best of our knowledge, this is the first study examining the relation between vitamin D and inflammation in neonates. The strengths of the study included prospective data collection, the large sample size, adjustment for essential confounders, and stratifications by 25(OH)D and birth season. However, the cross-sectional analysis in our study could not examine temporal relation and causality. One important limitation was the absence of data during October, November, and December, because the data collection was performed between January and September. However, the missing data was supposed to be divided into the winter-spring and summer-autumn. Consequently, we speculated that the absence of data might not substantially change the results of seasonality. Although pregnant women with acute inflammation have been excluded prior to sampling, residual confounding resulting from other covariates including maternal illness, chorioamnionitis, and fetal distress at birth could not be ruled out.

## 5. Conclusions

The evidence in our study indicated that cord blood CRP was inversely associated with vitamin D in neonates with low 25(OH)D levels or born in winter-spring. Neonates with vitamin D deficiency had higher risk of exposure to elevated inflammation at birth, which has been suggested to be associated with adverse health outcomes. However, fetal vitamin D levels in upper range might have no additional effects on the protection against inflammation. Clinically, for fetal vitamin D levels are largely dependent on maternal vitamin D status [40], the findings suggested that vitamin D supplements during pregnancy should be encouraged, especially for pregnant women with vitamin D deficiency or experiencing the winter-spring. Further interventional trials are needed to confirm the potential protective effect of adequate vitamin D on inflammatory response in a fetus/neonate.

## References

[1] Chen Y.H., Fu L., Hao J.H., Yu Z., Zhu P., Wang H., Xu Y.Y., Zhang C., Tao F.B., Xu D.X. (2015). Maternal vitamin D deficiency during pregnancy elevates the risks of small for gestational age and low birth weight infants in Chinese population. J. Clin. Endocrinol. Metab..

[2] Cizmeci M.N., Kanburoglu M.K., Akelma A.Z., Ayyildiz A., Kutukoglu I., Malli D.D., Tatli M.M. (2015). Cord-blood 25-hydroxyvitamin D levels and risk of early-onset neonatal sepsis: A case-control study from a tertiary care center in Turkey. Eur. J. Pediatr..

[3] Whitehouse A.J., Holt B.J., Serralha M., Holt P.G., Kusel M.M., Hart P.H. (2012). Maternal serum vitamin D levels during pregnancy and offspring neurocognitive development. Pediatrics.

[4] Amarilyo G., Oren A., Mimouni F., Ochshorn Y., Deutsch V., Mandel D. (2010). Increased cord serum inflammatory markers in small-for-gestational-age neonates. J. Perinatol..

[5] Franz A.R., Steinbach G., Kron M., Pohlandt F. (1999). Reduction of unnecessary antibiotic therapy in newborn infants using interleukin-8 and C-reactive protein as markers of bacterial infections. Pediatrics.

[6] Stolp H.B., Turnquist C., Dziegielewska K.M., Saunders N.R., Anthony D.C., Molnár Z. (2011). Reduced ventricular proliferation in the foetal cortex following maternal inflammation in the mouse. Brain.

[7] Zhang Y., Leung D.Y., Richers B.N., Liu Y., Remigio L.K., Riches D.W., Goleva E. (2012). Vitamin D inhibits monocyte/macrophage proinflammatory cytokine production by targeting MAPK phosphatase-1. J. Immunol..

[8] Almerighi C., Sinistro A., Cavazza A., Ciaprini C., Rocchi G., Bergamini A. (2009). 1α, 25-dihydroxyvitamin D3 inhibits CD40L-induced pro-inflammatory and immunomodulatory activity in human monocytes. Cytokine.

[9] Standage S.W., Wong H.R. (2011). Biomarkers for pediatric sepsis and septic shock. Expert Rev. Anti-Infect. Ther..

[10] Hofer N., Zacharias E., Müller W., Resch B. (2012). An update on the use of C-reactive protein in early-onset neonatal sepsis: Current insights and new tasks. Neonatology.

[11] Trochez-Martinez R., Smith P., Lamont R. (2007). Use of C-reactive protein as a predictor of chorioamnionitis in preterm prelabour rupture of membranes: A systematic review. BJOG.

[12] Romem Y., Artal R. (1984). C-reactive protein as a predictor for chorioamnionitis in cases of premature rupture of the membranes. Am. J. Obstet. Gynecol..

[13] Wersching H., Duning T., Lohmann H., Mohammadi S., Stehling C., Fobker M., Conty M., Minnerup J., Ringelstein E.B., Berger K. (2010). Serum C-reactive protein is linked to cerebral microstructural integrity and cognitive function. Neurology.

[14] Ridker P.M., Rifai N., Rose L., Buring J.E., Cook N.R. (2002). Comparison of C-reactive protein and low-density lipoprotein cholesterol levels in the prediction of first cardiovascular events. N. Engl. J. Med..

[15] Pradhan A.D., Manson J.E., Rifai N., Buring J.E., Ridker P.M. (2001). C-reactive protein, interleukin 6, and risk of developing type 2 diabetes mellitus. JAMA.

[16] Amer M., Qayyum R. (2012). Relation between serum 25-hydroxyvitamin D and C-reactive protein in asymptomatic adults (from the continuous national health and nutrition examination survey 2001 to 2006). Am. J. Cardiol..

[17] Jorde R., Sneve M., Torjesen P.A., Figenschau Y., Gøransson L.G., Omdal R. (2010). No effect of supplementation with cholecalciferol on cytokines and markers of inflammation in overweight and obese subjects. Cytokine.

[18] Schleithoff S.S., Zittermann A., Tenderich G., Berthold H.K., Stehle P., Koerfer R. (2006). Vitamin D supplementation improves cytokine profiles in patients with congestive heart failure: A double-blind, randomized, placebo-controlled trial. Am. J. Clin. Nutr..

[19] Bjorkman M., Sorva A., Tilvis R. (2009). C-reactive protein and fibrinogen of bedridden older patients in a six-month vitamin D supplementation trial. J. Nutr. Health Aging.

[20] Haddad J.G., Boisseau V., Avioli L.V. (1971). Placental transfer of vitamin D3 and 25-hydroxycholecalciferol in the rat. J. Lab. Clin. Med..

[21] Jaye D.L., Waites K.B. (1997). Clinical applications of C-reactive protein in pediatrics. Pediatr. Infect. Dis. J..

[22] Chiesa C., Signore F., Assumma M., Buffone E., Tramontozzi P., Osborn J.F., Pacifico L. (2001). Serial measurements of C-reactive protein and interleukin-6 in the immediate postnatal period: Reference intervals and analysis of maternal and perinatal confounders. Clin. Chem..

[23] Holmes V.A., Barnes M.S., Alexander H.D., McFaul P., Wallace J.M. (2009). Vitamin D deficiency and insufficiency in pregnant women: A longitudinal study. Br. J. Nutr..

[24] Yu X., Wang W., Wei Z., Ouyang F., Huang L., Wang X., Zhao Y., Zhang H., Zhang J. (2014). Vitamin D status and related factors in newborns in Shanghai, China. Nutrients.

[25] Society C.P. (2007). Vitamin D supplementation: Recommendations for Canadian mothers and infants. Paediatr. Child Health.

[26] Zhang B.L. (1992). Revised values of birth weight by gender and gestational age in 15 cities of China. Acta Pediatirc J..

[27] Ngo D.T., Sverdlov A.L., McNeil J.J., Horowitz J.D. (2010). Does vitamin D modulate asymmetric dimethylarginine and C-reactive protein concentrations?. Am. J. Med..

[28] Bellia A., Garcovich C., D’Adamo M., Lombardo M., Tesauro M., Donadel G., Gentileschi P., Lauro D., Federici M., Lauro R. (2013). Serum 25-hydroxyvitamin D levels are inversely associated with systemic inflammation in severe obese subjects. Intern. Emerg. Med..

[29] Robinson A.B., vin Tangpricha M., Eric Yow M., Reut Gurion D., Grace A. (2013). Vitamin D deficiency is common and associated with increased C-reactive protein in children with lupus: An atherosclerosis prevention in pediatric lupus erythematosus (APPLE) substudy. Lupus Sci. Med..

[30] Chen N., Wan Z., Han S.F., Li B.Y., Zhang Z.L., Qin L.Q. (2014). Effect of vitamin D supplementation on the level of circulating high-sensitivity C-reactive protein: A meta-analysis of randomized controlled trials. Nutrients.

[31] Asemi Z., Samimi M., Tabassi Z., Shakeri H., Esmaillzadeh A. (2013). Vitamin D supplementation affects serum high-sensitivity C-reactive protein, insulin resistance, and biomarkers of oxidative stress in pregnant women. J. Nutr..

[32] Del Valle H.B., Yaktine A.L., Taylor C.L., Ross A.C. (2011). Dietary Reference Intakes for Calcium and Vitamin D.

[33] Grant C.C., Stewart A.W., Scragg R., Milne T., Rowden J., Ekeroma A., Wall C., Mitchell E.A., Crengle S., Trenholme A. (2014). Vitamin D during pregnancy and infancy and infant serum 25-hydroxyvitamin D concentration. Pediatrics.

[34] Burris H.H., van Marter L.J., McElrath T.F., Tabatabai P., Litonjua A.A., Weiss S.T., Christou H. (2013). Vitamin D status among preterm and full-term infants at birth. Pediatr. Res..

[35] Woodhouse P., Khaw K., Plummer M., Meade T., Foley A. (1994). Seasonal variations of plasma fibrinogen and factor VII activity in the elderly: Winter infections and death from cardiovascular disease. Lancet.

[36] Sung K.C. (2006). Seasonal variation of C-reactive protein in apparently healthy Koreans. Int. J. Cardiol..

[37] Horan J.T., Francis C.W., Falsey A.R., Kolassa J., Smith B.H., Hall W.J. (2001). Prothrombotic changes in hemostatic parameters and C-reactive protein in the elderly with winter acute respiratory tract infections. Thromb. Haemost..

[38] Liu P.T., Stenger S., Li H., Wenzel L., Tan B.H., Krutzik S.R., Ochoa M.T., Schauber J., Wu K., Meinken C. (2006). Toll-like receptor triggering of a vitamin D-mediated human antimicrobial response. Science.

[39] Mellenthin L., Wallaschofski H., Grotevendt A., Völzke H., Nauck M., Hannemann A. (2014). Association between serum vitamin D concentrations and inflammatory markers in the general adult population. Metabolism.

[40] ACOG (2011). Vitamin D: Screening and supplementation during pregnancy. ACOG Committee Opinion No. 495. Obstet. Gynecol..

